# Supplementary material for: Comparison Study of an Optimized Ultrasound-Based Method versus an Optimized Conventional Method for Agar Extraction, and Protein Co-Extraction, from Gelidium sesquipedale
Source: Foods. 2022 Mar 11;11(6):805. doi: 10.3390/foods11060805 (PMC8947469; doi:10.3390/foods11060805)
Supplement: Supplementary file 1 [file foods-11-00805-s001.zip › foods-1614423-supplementary.pdf]

Energy measurement by using a power meter

The price of the energy required to extract agar by the optimized conventional extraction method and the optimized UAE was calculated by pre-setting the price of energy on 1 €/ kW·h in a power meter. The systems employed were connected to a power meter which was plugged to the electric supply. The energy usage was measured twice per experiment (R1: repetition 1, and R2: repetition 2), except in the case of the ultrasound probe, only measured once.

Experiment	Cost of energy in Euros
Optimized conventional extraction (6 h, 110 °C) R_1	16.54 (oil bath)
Optimized conventional extraction (6 h, 110 °C) R_2	20.29 (oil bath)
UAE optimized extraction (1 h, 100% power) R_1	9.53 (water bath)
UAE optimized extraction (1 h, 100% power) R_2	4.90 (water bath) 1.0 (US probe)
